# Melatonin decreases estrogen receptor binding to estrogen response elements sites on the OCT4 gene in human breast cancer stem cells

**DOI:** 10.18632/genesandcancer.107

**Published:** 2016-05

**Authors:** Juliana Lopes, David Arnosti, James E. Trosko, Mei-Hui Tai, Debora Zuccari

**Affiliations:** ^1^ Department of Biology, Universidade Estadual Paulista “Júlio de Mesquita Filho”, São José do Rio Preto, SP, Brazil; ^2^ Department of Biochemistry and Molecular Biology, Michigan State University, East Lansing, MI, USA; ^3^ Department of Pediatrics and Human Development, Michigan State University, East Lansing, MI, USA; ^4^ Department of Molecular Biology, Faculdade de Medicina de São José do Rio Preto, São José do Rio Preto, SP, Brazil

**Keywords:** melatonin, estrogen receptor, chromatin immunoprecipitation, three-dimensional growth, mammospheres

## Abstract

Cancer stem cells (CSCs) pose a challenge in cancer treatment, as these cells can drive tumor growth and are resistant to chemotherapy. Melatonin exerts its oncostatic effects through the estrogen receptor (ER) pathway in cancer cells, however its action in CSCs is unclear. Here, we evaluated the effect of melatonin on the regulation of the transcription factor OCT4 (Octamer Binding 4) by estrogen receptor alpha (ERα) in breast cancer stem cells (BCSCs). The cells were grown as a cell suspension or as anchorage independent growth, for the mammospheres growth, representing the CSCs population and treated with 10 nM estrogen (E2) or 10 μM of the environmental estrogen Bisphenol A (BPA) and 1 mM of melatonin. At the end, the cell growth as well as OCT4 and ERα expression and the binding activity of ERα to the OCT4 was assessed. The increase in number and size of mammospheres induced by E2 or BPA was reduced by melatonin treatment. Furthermore, binding of the ERα to OCT4 was reduced, accompanied by a reduction of OCT4 and ERα expression. Thus, melatonin treatment is effective against proliferation of BCSCs *in vitro* and impacts the ER pathway, demonstrating its potential therapeutic use in breast cancer.

## INTRODUCTION

Approximately 80% of breast tumors are classified as ER positive and is correlated with better prognosis and a greater response to hormonal therapy [1]. ER positive tumors use steroid hormone, estradiol [E2], as their main growth stimulant, therefore ER is the direct target of endocrine therapies [2]. Many chemicals can mimic the biological functions of E2, contributing to the initiation or progression of cancer [3]. These environmental estrogens include Bisphenol A (BPA) is widely used in industry for the manufacture of polymers such as polycarbonate and films used in food containers [4]. BPA activates the transcription of genes responsible for the increase in cell proliferation of the breast cancer cells [5]. In light of high rates of breast cancer, it has become vital to understand the action of new drugs that might be effective against this disease.

The melatonin hormone appears to have an oncostatic effect in differenttypes of cancers [5, 6]. This effect is seen on hormone-dependent tumors, and its action has greater relevance in breast tumors [7]. A suggested mechanism was that melatonin decreases transcription of the *ER* gene in MCF-7 cells, leading to reduced levels of the ER-estrogen complex binding targets in the genome [8–10]. However, the action of melatonin on the ER pathway has not been well studied in cancer stem cells, especially for breast cancer stem cells (BCSCs) [11–12]. Such cancer stem cells are able to re-initiating tumor growth, being responsible for tumor recurrences, metastasis and drug resistance [13]. From studies of cancer stem cells, the transcription factor OCT4, encoded by the *POU5F1* gene, was found to be a critical factor for self- renewal and maintenance of pluripotency of stem cells [14, 15]. Based on the idea that breast cancer treatment is particularly complicated by cancer stem cells, and that the control of the disease requires the inhibition of these cells, the objective of this study was to evaluate the effect of melatonin on regulation of the *OCT4/POU5F1* by the ERα in BCSC after induction with tumor initiation chemical BPA and with E2.

## RESULTS

### Effect of melatonin on cell suspension and anchorage independent growth (AIG)

**Figure 1:**
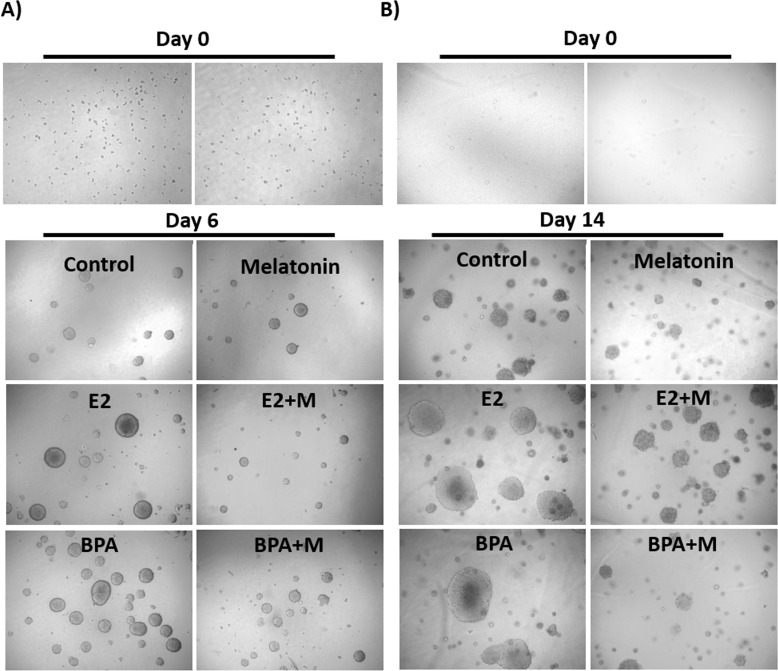
MCF-7 cells grown in 3-dimensional method of mammospheres, treated with E2 or BPA with or without melatonin (A) Cell suspension treated for 6 days. (B) Anchorage Independent Growth (AIG) treated for 14 days. The magnification was 40 X.

**Figure 2:**
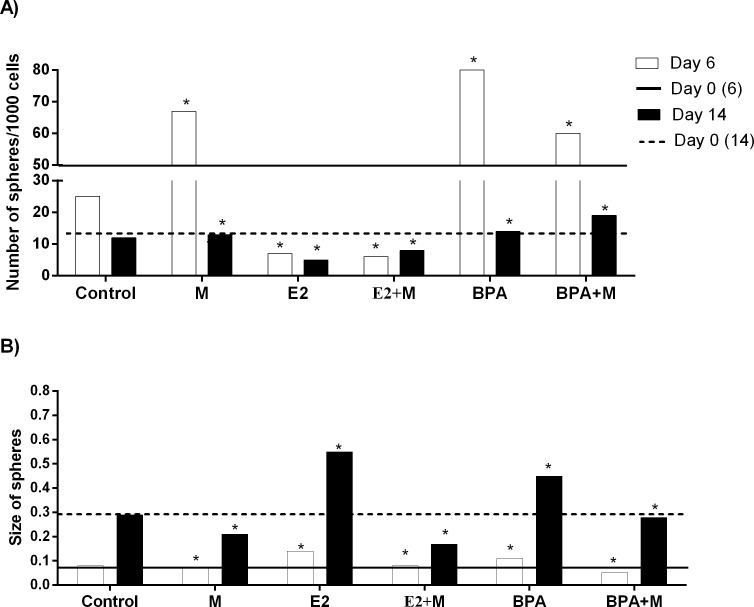
Effect of E2 or BPA with or without melatonin on MCF-7 mammospheres (A) Number of mammospheres. (B) Size of mammospheres. Significant value in ANOVA followed by Bonferroni's test (*P ≤ 0.05).

To evaluate the effect of melatonin on mammospheres treated with BPA and E2, two methods of 3-dimensional growth were performed (cell suspension and AIG). For the cell suspension and AIG technique, the results were similar (Figure 1A, 1B). The treatment with 10 nM E2 and 10 μM BPA without melatonin significantly increased the number and the size of the mammospheres when compared with the control group (Figure 2A, 2B) in both technique. On the other hand, 1 mM of melatonin significantly decreased the number and size of mammospheres when compared with the control (Figure 2A, 2B). Furthermore, when the cells were stimulated by E2 or BPA and treated with melatonin concomitantly, there was a greater reduction in the number and size of mammospheres (Figure 2A, 2B).

### Effect of melatonin on interaction of estrogen receptor with *POU5F1* gene

**Figure 3:**
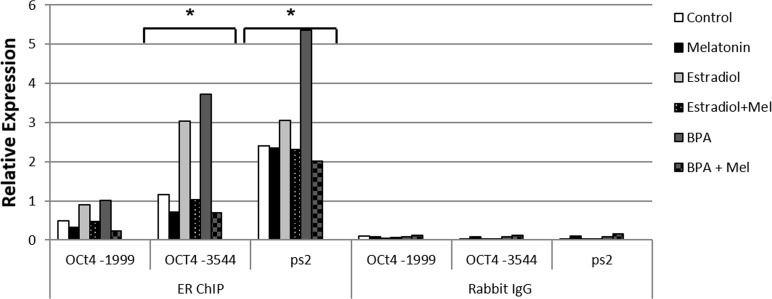
Chromatin immunoprecipitation to verify the binding activity of ER to the putative ERE sequences in OCT4 transcription site (OCT4 -1999 and OCT4 -3544) after treatment with E2 or BPA with or without melatonin in mammospheres Significant value in ANOVA followed by Bonferroni's test (*P ≤ 0.05).

In order to analyze the effect of melatonin on estrogen receptor binding to the *POU5F1 gene,* we carried out the Chromatin Immunoprecipitation assay. The results showed that mammospheres treated with 10nM E2 significantly increased the binding of ER to ERE sites at −3544 kb of OCT4 promoter region with similar level when compared to pS2, and the same occurred when treated with 10μM BPA (Figure 3). On the other hand, the cells stimulated by 10nM E2 or 10μM BPA and treated with 1mM melatonin showed decreased binding of ER on OCT4 promoter (Figure 3). The another putative binding site at −1999 kb showed a slight increase of ER binding, when treated with E2 or BPA, however, the enhancement of binding level was very low and when treated with melatonin this enhancement was decreased (Figure 3).

### Impact of melatonin on transcription of the ER and *POU5F1* genes

**Figure 4:**
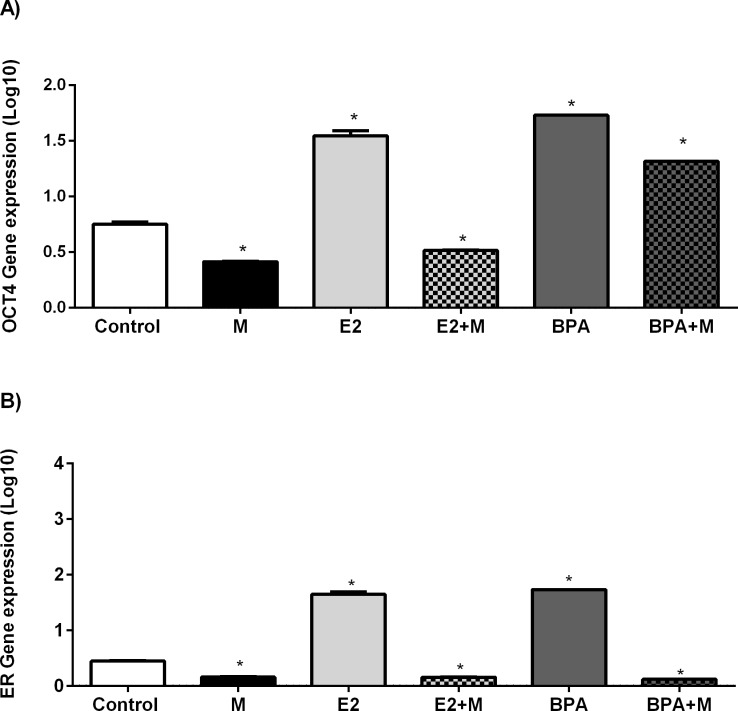
Analysis of OCT4 and ER gene expression after treatment with melatonin, E2 and BPA (A) OCT4 gene expression. (B) ER gene expression. The gene expression values were log10 represented. Significant value in ANOVA followed by Bonferroni's test (*P ≤ 0.05).

In order to examine the effects of melatonin on transcription of the *ER*α and *POU5F1*, the expression of these genes were measured. As expected, cells treated with E2 or BPA exhibited increased levels of transcript for OCT4 and ERα (Figure 4A, 4B). Melatonin treatment had the opposite effect; levels of OCT4 and ERα mRNA were sharply decreased in cells treated with melatonin alone, or in combination with either E2 or BPA (Figure 4A, 4B).

### Impact of melatonin on expression of ER and OCT4 proteins

**Figure 5:**
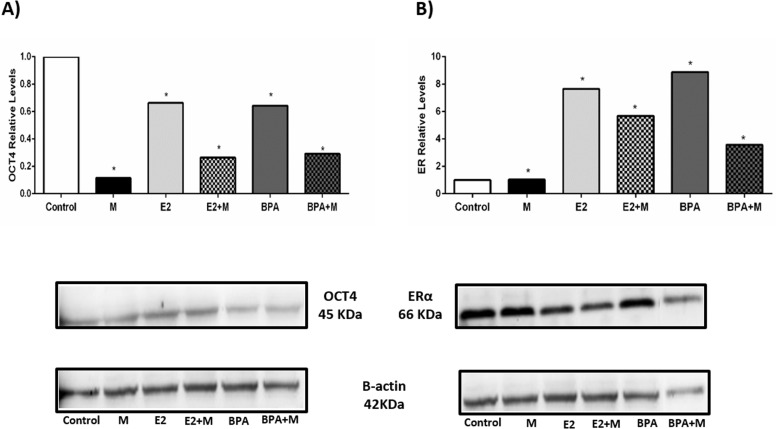
Analysis of OCT4 and ER protein expression after treatment with melatonin, E2 and BPA (A) OCT4 protein expression. (B) ER protein expression. Protein expression values were represented in relative levels. OCT4 and ER protein levels were normalized to β-actin protein, shown in the boxes. Significant value in ANOVA followed by Bonferroni's test (*P ≤ 0.05).

Also, we tested the impact of estrogen, BPA and melatonin on ER and OCT4 protein levels in these cells. E2 and BPA significantly raised levels of ERα protein (Figure 5B). Melatonin alone had no discernible impact on protein levels, despite the decrease in transcript level noted above. ERα protein levels were generally decreased in all cells treated with melatonin, but the extent of decrease did not closely mirror the changes in mRNA levels, suggesting that additional levels of regulation may be impacting ERα levels. Melatonin similarly decreased levels of OCT4 protein (Figure 5A); the decrease was quite significant in cells treated with melatonin alone, where no significant change in ERα protein levels had been noted (Figure 5B). Only slight changes in OCT4 transcript levels had been observed, suggesting that regulation of protein levels of OCT4 may be controlled at a translational and/ or post-translational level. OCT4 protein levels were also increased in cells treated with E2 or BPA (Figure 5A), although not as dramatically, thus the elevated levels of ERα may contribute to increased expression of OCT4 protein through the transcriptional effects noted above.

## DISCUSSION

The purpose of our study was to evaluate the melatonin effect after the induction with tumor initiation chemical BPA and E2 in MCF-7 cells using two different techniques of three-dimensional growth of mammospheres (cell suspension and AIG), representing the CSCs population. The growth in a three-dimensional (3D) model creates an artificial tumor environment where the cells segregate properly to form components of adult tissues analogous to the corresponding found *in vivo* [2]. This type of cell culture can be used to detect agents that affect the growth of breast cancer stem cells [2]. Our results demonstrated that the treatment with 10nM E2 or 10μM BPA increased the number and the size of the mammospheres in both technique. On the other hand, 1mM melatonin decreased the number and size of mammospheres treated with 10nM E2 or 10μM BPA. It was suggested the involvement of E2 and BPA in the initiation and progression of breast cancer, increasing the cell proliferation rate [17]. MCF-7 BCSCs treated with E2 and BPA, showed an increase in cell proliferation rate [18, 19].

Estrogen exposure has been strongly linked to breast cancer risk. Women who have had hormone replacement therapy with estradiol have an increased risk of develop breast tumors, consistent with the established relationship between estrogen and breast cancer [20]. Environmental factors may similarly impact the risk of breast cancer; for instance, the estrogenic actions of BPA have been shown in studies where the compound activated estrogen receptors and stimulating the growth of MCF-7 breast cancer cells [21, 22]. From these studies, it is has been suggested that this compound may play a role in the initiation of breast cancer. Conversely, melatonin has been shown to inhibit the proliferation of estrogen-responsive MCF-7 human breast cancer cells [23]. Cos et al. [24] demonstrated the action of melatonin in BCSC, the authors verified that melatonin treatment decreased the number and size of mammospheres. Melatonin administration has also been reported to block estradiol induced proliferation of MCF-7 cells [25, 26]. However, no studies have examined the possible effect of melatonin on breast cancer stem cells that were simultaneously treated with with BPA and E2, as might be expected to happen in natural settings.

We find that melatonin antagonizes the effects of estrogen and BPA; previous studies indicated that this hormone reduces ER binding activity. To determine whether these effects may influence stem cell growth through regulation of the *POU5F1* gene, we looked at direct regulation of the gene by ERα. We found increased binding of ERα to ERE sites of the *POU5F1* promoter region in mammospheres treated with E2 or BPA. This binding was reduced upon melatonin treatment. OCT4, the product of *POU5F1*, controls the self-renewal and pluripotency of stem cells and is expressed in germ cells, embryonic stem cells and human stem cells [27, 28]. Our findings are similar to those of Jung et al. [2], who also showed that MCF-7 mammospheres treated with E2 exhibited increased the binding of ERα to the ERE site of *POU5F1* at −3544 kb. However, in contrast to our findings, BPA has not changed the binding activity, suggesting that act through different mechanisms on the MCF-7 cells. Rato et al. [25] observed that MCF-7 cells stimulated with estradiol have a high binding activity of estradiol-estrogen receptor complexes (E2-ER) to the estrogen-responsive elements (ERE) in the DNA, which was inhibited by treatment with melatonin. Assuming that melatonin decreased the binding of estradiol to the estrogen receptor, impeding the complexes (E2-ER) formation and, consequently, the binding to the ERE in the DNA. Woo et al. [29] in 2001, also suggested that melatonin suppressed the action of E2, preventing estradiol binding to the estrogen receptor, confirming the participation of melatonin in the estrogen signaling pathway.

In our study, we showed that E2 or BPA treatment upregulated OCT4 and ERα transcript and protein levels. Concomitant treatment with melatonin reduced OCT4 and ERα transcript and protein expression to varying degrees. The action of melatonin on the ER pathway was reported Kiefer et al. [30], the MCF-7 cells were transfected with anestrogenresponse element (ERE) and treated withmelatoninand E2. They demonstrated that melatonin decreases ERα mRNA and protein levels. Therefore, it is suggested that melatonin acts in the ER levels, decreasing its activity.

The effect of melatonin on the OCT4 via was reported in one study [12], however, there are no studies that evaluated the action of melatonin on OCT4 in cancer stem cells. In 2011, Yoo et al. [12] demonstrated that melatonin did not change the expression of OCT4 gene in murine embryonic stem cells. Kannen et al. [11] demonstrated that treatment with melatonin in rats with colon cancer induced by a carcinogen 1,2-dimethylhydrazine, reduced cell proliferation and induce apoptosis of colon cancer cells, possibly through inhibition of CD133 (+), a glycoprotein which is expressed on tumor stem cells. OCT4 expression is important to the carcinogenesis process and its action may allow cancer cells to acquire or maintain the chemotherapy resistant phenotype.

In conclusion, our results show that melatonin counteracts the effects of E2 and BPA treatment on mammosphere growth as well as the expression of ERα and the stem cell marker OCT4. Our data suggests that the effect of melatonin on *POU5F1* (OCT4) may be in part a reflection of its impact on expression and activity of the estrogen receptor in BCSC. Due to the highly aggressive potential of cancer stem cells and of their resistance to cancer therapies, it is therefore of great interest to pursue the study of new drugs that act directly on this tumor population. These studies, in addition, provide a mechanistic explanation for the epidemiological observations on the risk to breast cancers in the absence of melatonin due to the loss of sleep at night. Our findings of the action of melatonin are therefore relevant to this field of cancer studies.

## MATERIALS AND METHODS

### Cell culture

The breast cancer cell line MCF-7 was obtained from ATCC (American Type Culture Collection, Manassas, VA, USA). The cells were grown at 5 % CO_2_ at 37°C in DMEM (E) (Invitrogen, Carlsbad, CA) with 10 % Fetal Bovine Serum (FBS), penicillin (100 IU/mL) and streptomycin (100 mg/mL). When the cells reached 90% - 100% confluence, were grown in two methods of 3-dimensional mammospheres, cell suspension and Anchorage Independent Growth (AIG).

The treatment with melatonin in both methods was done according to [16] and treatment with E2 and BPA was according to [2].

### Cell suspension

A total of 1 × 10^4^ cells diluted in 2 ml of DMEM (E) were plated on top of 2 ml of prehardened 1% agarose diluted in Phosphate-buffered saline (PBS) in each well of 6 wells plates. Simultaneously, the cells received the treatments for 6 days according to the following groups: I. Control (treated with vehicle); II. 1 mM Melatonin (M) (Sigma-Aldrich, St. Louis, MO, USA); III. 10 nM Estradiol (E2) (Sigma-Aldrich, St. Louis, MO, USA); IV. E2+M; V. 10 μM Bisphenol A (BPA) (Sigma-Aldrich, St. Louis, MO, USA) and group VI. BPA+M.Melatonin. BPA and E2 were diluted in ethanol 100%. In control cells, equivalent amount of ethanol was added as vehicle. At the end, the number and size of the MCF-7 mammospheres were measured. These treatment conditions were used for Chromatin immunoprecipitation (ChIP), quantitative RT- PCR analysis (qPCR) and western blotting.

### Anchorage Independent Growth

A total of 5 × 10^4^ cellsdiluted in 3 ml of 0.33% agarosemedium were grown on topof 3 ml of prehardened 0.5% agarosemedium in each of triplicate dishes (6 cm). Simultaneously, the cell treatment was performed for 2 weeks according to the following groups: I. Control (treated with vehicle); II. 1 mM of Melatonin (M); III. 10 nM Estradiol (E2); IV. E2+M; V. 10 μM Bisphenol A (BPA) and VI. BPA+M. Melatonin, BPA and E2 were diluted in ethanol 100%. In control cells, equivalent amount of ethanol was added as vehicle. At the end, the number and size of the MCF-7 mammospheres were scored.

### Chromatin Immunoprecipitation (ChIP)

To verify if ER bound to ERE sites on OCT4 promoter region, the ChIP assay was performed after E2 and BPA addition, with or without melatonin. For the assay, 5 × 10^6^ MCF-7 mammospheres were grown in cell suspension and treated with 10 μM BPA and 10 nM E2 with or without 1 mM melatonin for 6 days. After 6 days, cells were crosslinked with 1% formaldehyde for 30 min. Then, cells were pelleted and washed sequentially in PBS (pH 7.4), buffer I (10 mM HEPES [pH 6.5], 10 mM EDTA, 0.5 mM EGTA, 0.25% Triton X-100) and buffer II (10 mM HEPES [pH 6.5], 1 mM EDTA, 0.5 mM EGTA, 150 mM NaCl. The pellet was then suspended in lysis buffer (50 mM Tris, pH 8.0, 10 mM EDTA, 1% sodium dodecyl sulfate [SDS], 0.5 μM phenylmethylsulfonyl fluoride, 1 μM pepstatin A, 1 mM sodium bisulfite, 1 mM benzamidine, 1 mM dithiothreitol). Cells were sonicated seven times 10 pulses each round in lysis buffer using a Branson Sonifier 450D with microtip (Branson, Danbury, CT). After sonication, ChIPs were performed using anti- ERα (sc-8002. SantaCruz Biotechnologies) or rabbit non specific IgG (Santa Cruz Biotechnology) overnight at 4°C. Immunoprecipitations were performed with ChIP dilution buffer (20 mMTris [pH 8.0], 2 mM EDTA, 1% Triton X-100, 0.5 μM phenylmethylsulfonyl fluoride, 1 mM DTT) containing 150 mM NaCl. 40 ul of Protein A-agarose beads were then added, and the mixture was rocked for 4 h at 4°C. After a brief centrifugation, the immunoprecipitated material was washed sequentially in TSE buffer (20mMTris, pH8.0, 0.1% SDS, 2 mM EDTA, 1% Triton X-100), TSE buffer plus 250 mM NaCl, and TSE buffer plus 500 mM NaCl and in buffer III (10 mM Tris, pH 8.0, 1 mM EDTA, 0.25 M LiCl, 1% NP-40,1% deoxycholate). Beads were then washed in TE buffer (20 mM Tris, pH 8.0, 2 mM EDTA), and protein complexes were eluted in 300 μl of 0.1 M NaHCO_3_, 1% SDS. Cross- links were reversed overnight at 65°C. qRT-PCR was performed on the DNA extracts for ER, Input and IgG control using SYBR Green Master Mix reagents with an ABI 7500 sequence detection system. Enrichment of ERα at target genes promoters was examined using the following primers: OCT4 -3544 forward (TCC TCC CAG CTC ACC CAC TCC), OCT4 -3544 reverse (TCT CCC CCA TGA GCC CTG CA), OCT4 -1999 forward (GAC AGC TGG CCA CGG GAC AC) and OCT4 -1999 reverse (AGG CCA GGT CTG GAC TGG GC). As positive control, we used a well-characterized ER binding site on the *pS2* promoter: pS2 forward (CCT GCA AGG TCA CGG TGG CC) and pS2 reverse (GGC CCT CCC GCC AGG GTA AA).

### RNA Extraction and Gene Expression Analysis Using Quantitative Real Time PCR (qPCR)

To verify the OCT4 and ERα expression levels after E2 and BPA treatment with or without melatonin, we performed qPCR. For the assay, 0.5 × 10^5^ MCF-7 mammospheres were grown in cell suspension and treated with 10 μM BPA and 10 nM E2 with or without 1 mM melatonin for 6 days. After 6 days, total RNA was extracted from the mammospheres using trizol reagent and the cDNA was synthesized using Super Script II Reverse Transcriptase (Invitrogen) in a total volume of 20 μl.

The quantitative expression was performed through RT-PCR in triplicate, using Step OnePlus System (Applied Biosystems, Foster City, CA, USA) and a negative control was included in each reaction. PCR reactions contained 4 μl of cDNA (100 ng), 10 μl of SYBR Green Master Mix, 4 μl of DEPC water and 2 μl of primers for OCT4, ERα and ACTB were performed. Primers sequences were as follows: ERα- F, CGT CGC CTC TAA CCT CGG, ERα- R, CCC AGA TGC TTT GGT GTG GA, OCT4- F, CTC TGC AGA TTC TGA CCG CA, OCT4- R, CAT GGG TGA GGG TAG TCT GC and ACTB – F, CAC AGA GCC TCG CCT TTG C and ACTB – R, GCG CGG CGA TAT CAT CAT CC. The levels of mRNA of the *POU5F1* and *ER* genes were normalized to *ACTB* gene as an internal control of expression. The relative gene expression was measured by the ΔΔCt method. The value of Relative Quantification (RQ) of the control group (used as reference) was established as the unit for analysis of the expression of OCT4 and ER.

### Western blotting

To measure the effect of E2, BPA and melatonin treatment on OCT4 and ER protein expression, 5 × 10^5^ MCF-7 mammospheres were grown in cell suspension and treated with 10 μM BPA and 10 nM E2 with or without 1 mM melatonin for 6 days. After 6 days, mammospheres were harvested and the pellet was snap- frozen in liquid nitrogen. Cell extracts were thawed in lysis buffer (50 mM Tris HCl, pH 8.0, 150 mM NaCl, 1% Triton X-100) and centrifuged. The supernatant was used in Bradford assays and Western blot assays. Equal amounts of whole cell extracts (50 μg) were separated by 12.5% SDS-PAGE and transferred to nitrocellulose membranes for Western analyses. Endogenous proteins were detected by using the following antibodies: anti- OCT4 (ab27985, mouse polyclonal, 1: 1 000, Abcam), anti-ERα (SC-543, mouse polyclonal, 1: 1 000 SantaCruz Biotechnologies) and anti-β-actin (sc-47778, mouse monoclonal, 1: 5 000 SantaCruz Biotechnologies). The antibody incubation was performed in 5% milk in TBST (20 mM Tris HCl, pH 7.5, 120 mM NaCl, 0.1% Tween20). Blots were developed using peroxidase-conjugated goat anti-mouse secondary antibodies as appropriate (1: 5 000, ThermoScientific, Waltham, MA) and SuperSignal WestPico Chemiluminescent substrate (ThermoScientific). Quantification was performed using Image J software as image analyzer.

### Statistical analysis

All results were submitted to descriptive analysis to determine statistical normality. An analysis of variance (ANOVA) was performed, followed by the Bonferroni's test. Values of P ≤ 0.05 were considered statistically significant. The GraphPad Prism 6 software (GraphPad Software, inc., San Diego, CA, USA) was used.
